# Exposure to a musically-enriched environment; Its relationship with executive functions, short-term memory and verbal IQ in primary school children

**DOI:** 10.1371/journal.pone.0207265

**Published:** 2018-11-12

**Authors:** Artur C. Jaschke, Henkjan Honing, Erik J. A. Scherder

**Affiliations:** 1 Department of Clinical Neuropsychology, VU University Amsterdam, Amsterdam, the Netherlands; 2 Department of Music Therapy, ArtEZ University of the Arts, Enschede, the Netherlands; 3 Music Cognition Group, Amsterdam Brain and Cognition, Institute for Logic, Language and Computation, University of Amsterdam, Amsterdam, the Netherlands; University of Zurich, SWITZERLAND

## Abstract

**Background:**

Previous literature has shown a putative relationship between playing a musical instrument and a benefit in various cognitive domains. However, to date it still remains unknown whether the exposure to a musically-enriched environment instead of playing an instrument yourself might also increase cognitive domains such as language, mathematics or executive sub-functions such as for example planning or working memory in primary school children.

**Design:**

Cross-sectional

**Method:**

Exposure to a musically-enriched environment like listening to music at home, during play or when attending concerts was assessed using a comprehensive intake questionnaire administered to a sample of 176 primary school children. Furthermore, participants completed the verbal intelligence section of the Wechsler Intelligence Scale (WISC III), performed executive sub-function tasks such as planning (Tower of London), working memory (Klingberg Matrix backward span) and inhibition (Go/no-Go task), and a short-term memory task (Klingberg Matrix forward span).

**Results:**

Linear and multiple regression analyses showed no significant relationship between exposure to a musically-enriched environment, executive sub-functions (planning, inhibition and working memory), and short-term memory. The relationship between an enriched musical environment and verbal IQ has revealed trends.

**Discussion:**

Experiencing a musically enriched environment does not serve as predictor for higher performance on executive sub-functions, however, can influence verbal IQ.

## Introduction

Scientific interest into the possible effects of music on cognitive functions has increased considerably over the last decades [1–7]. More specifically, the applications of music interventions such as instrumental or vocal lessons have been examined in relation to cognitive functions such as intelligence [1,2], mathematics and language [8,9]. These results, however, remain equivocal.

Regarding the effects of music lessons on mathematical abilities, Rickard, Bambrick and Gill [10] have shown a positive effect, while Costa-Giomi [11] could not support a positive effect of music education on mathematical abilities. Similar split results were found in studies analysing language skills, i.e. negative effects on writing [12] and positive relationships to reading [13,14]. These findings thus indicate that a ‘far transfer’ effect of music on a variety of cognitive functions is not consistent. The question arises as to how to explain these equivocal results in light of overall skills such as mathematics or reading.

As argued by Barnett and Ceci [15], processing models of far transfer account for differences in the individual processes of domain-related cognitive skills. This supports the idea of dividing academic skills into building blocks, such as application, operation and execution in, for example, mathematics. Far transfer, therefore, can only be reliably demonstrated when these subdivisions, both in the execution of cognitive skills and in the analysis of the sub domain of far transfer as proposed by Barnett and Ceci [15], are considered and analysed individually.

Furthermore, a recent meta-analysis [16] has stressed the difficulty of finding a transfer effect from music education to cognitive and academic skills in children completely. The authors state that most studies have neither used randomisation of participants, nor described the intervention and methods thoroughly enough to be able to draw clear conclusions. They argue furthermore that the use of active control groups often lacks in studies investigating a possible far transfer effect from music to cognitive skills. Schlaug [17] and Swaminathan, Schellenberg and Khalil [18] have argued similarly, urging future research into the relationship of music and cognitive skills to use thorough participant randomisation and longitudinal analysis, to eliminate factors such as home support, socio-economic differences or musical aptitude.

We argue that the focus of existing studies on cognitive functions such as language and mathematics is one step too far. Perhaps, one should examine whether music has a more profound effect on functions underlying language and mathematics, such as executive sub-functions (EsF) [19,20]. EsF include planning, set-shifting, working memory, attention and impulse control [21]. Indeed, executing a verbal mathematical task requires functions such as working memory, attention, inhibition, and planning [22,23], i.e. functions that overlap when perceiving and processing music [24].

Against this backdrop, the study of influence of music interventions on general cognitive abilities such as EsF and academic achievement depends on the definition of music intervention itself. Music interventions encompass active music making [25], active listening to music [26] or exposure to music, as in passive music listening. Unfortunately, not all children play a musical instrument, but they may be exposed to a musically-enriched environment (MEE) to varying extents. Being exposed to a MEE can be defined as an environment where more than just standard music lessons are provided to the child. It includes attending concerts outside of the school curriculum, listening to music at home during the execution of certain tasks, and singing while playing or interacting with peers.

All these contribute to an enriched environment with the child participating actively, without effort, thus enjoying these activities [27,28,29]. Our interest is in whether ‘just’ being exposed to a musical environment in a home and peer-to-peer setting might be sufficient to find a relationship with higher cognitive functions such as EsF, short-term memory, and verbal intelligence. Therefore, the goal of the present study is to examine if exposure to a MEE (i.e. listening, attending concerts, interacting with music outside of school) is related to specific cognitive functions, including EsF, short-term memory, and verbal IQ, in primary school children.

## Method

### Participants

The sample comprised 176 third and fourth grade children recruited from five different primary schools in the Netherlands, consisting of 74 (39%) boys and 102 (61%) girls who were matched for handedness (N = 15 girls and N = 8 boys). Ages ranged from 5 years and 10 months to 8 years and 10 months, with a mean age of 6 years and 10 months (SD = 7 months) Table 1 shows all participant characteristics (Table 1).

**Table 1:** Participant characteristics.

	*Total* *(n = 176)*		Test statistics	
*Demographics*	mean	SD	t	p <
MME (0–25)	13.5	5.9	- 5.32	0.01
Age (years. months)	6. 10	0.7	1.61^a^	0.784
Gender (% girls)	61		2.01^a^	0.14
*Parents education level*				
Mother	4.31^b^		0.22	0.91
Father	3.96^b^		0.13	0.78
*Executive sub-function scores*				
ToL	26.7	2.367	1.33^b^	0.42
Go/noGO	.468	0.264	0.15^b^	0.62
Klingberg WM	5.48	3.105	0.67^b^	0.25
*Other cognitive functions*				
Klingberg StM	7.33	3.167	0.75^b^	0.73
WISC-III	111.4	2.764	0.34^b^	0.02

Notes: MME = Musically Enriched Environment; ToL = Tower of London Baseline scores, Go/noGo = inhibition baseline scores; WM = working memory baseline scores; StM = short term memory baseline scores; WISC-III = Wechsler Intelligence Scale for Children 3^rd^ Edition total baseline sub-tests scores

^a^ = χ^2^ test

^b^ scores represent educational levels above basic secondary school

### Design

The present cross-sectional study includes baseline data from a longitudinal block randomisation-controlled trial examining the effect of music education on EsF, short-term memory, and verbal IQ in primary school children (for details see [24]). Baseline data included in the present study, represent the first measure of all cognitive tests before administering the music intervention in our longitudinal follow-up measure.

### Procedure

Trained research assistants administered each test, whereby each participant was tested individually in a quiet environment during school hours. Total testing time was 1.5 hours per participant, thereby minimising disturbance during school hours as much as possible.

The whole test protocol was administered in one session with short breaks where necessary to motivate and allow the participants to regain their focus on the tasks. All tasks were presented in a child-friendly manner and aimed at creating a ‘computer-game’ environment.

### Exclusion criteria

We have used the following exclusion criteria: inability to perform neuro-psychological testing due to dyslexia, dyscalculia, severe deafness and blindness or insufficient motor command of both arms.

### Medication

Nine participants use medication. However, the medication was not associated with a possible decrease in cognitive performance (Zomacton, Movicolon, Ventolin, Zyrtec, Aerius, D-Amo-X.Z., Broxil and Flixotide).

### Informed consent

Informed consent was obtained prior to the research from the child’s parents or legal representatives. The Medical Ethical Committee of the VU University Medical Centre Amsterdam as well as the Research and Ethics committee of the VU University Amsterdam have approved this study.

### Materials

#### Intake questionnaire

Parents were asked to complete a self-created intake questionnaire which included questions about socio-economic background as measured by level of education, health issues of their child and the general home situation e.g. interaction with siblings, behaviour at home such as calm, angry at times or violent, within general domestic challenges. Socio-economic status was estimated by each parents’ education levels individually. Education levels were distributed equally across the subgroups with no parent scoring lower than basic secondary school education.

An important part of this intake questionnaire investigated general exposure to music, reflected in, for example, concert attendance, musical engagement at home as well as formal music lessons and their duration and frequency outside of school (children have not yet received music lessons at school at time of the baseline measure). Moreover, parents’ values of a musical environment at home was analysed by asking questions as to what music is played, how often and in which context (leisure, learning or other). Furthermore, a full-scale analysis of exposure, frequency and parents’ musical aptitudes was conducted through the intake questionnaire yielding a score for a MEE (Table 2).

**Table 2:** MEE, excerpt from the self-created intake questionnaire.

Survey Theme	Question summaries	Scoring
Music lessons	*Does the child receive music lessons*	Q [0,1]
	private at school both	
	*Type of music lessons* individual group	Q [0,1]
	length 15–30 min 30–45 min 45–60 min 60 + min (please specify)	Q [0,1]
	which instrument does your child play (please specify)	D
Music preferences	which genre/sort of music (please specify)	D
Music exposure	when does the child listen/sing at home social with peers during homework during play	Q [0,1] [0,1] [0,1] [0,1] [0,1]
	average length of exposure per ‘session’ (please specify)	D
Music activity	child puts on music him/herself	Q [0,1]
	eength of listening	D
Parents use of music	exposing the child to music: at home concerts during tasks / homework / play	Q [0–5] [0–5] [0–5]
	Mother / Father is musician active amateur musician none	Q [0,1]

Excerpt of intake questionnaire on quantification of a *musically enriched environment*. D = Descriptive: information was filled into the questionnaire by the parent or legal representative, Q = Quantitative: groupings were made e.g. time practised or time exposure to music for quantification, being scored with 0 = No and 1 = Yes. Additionally, exposing the child to music was assessed with a Likert scale (0–5); 0 *little to no exposure*, 5 *a lot of exposure*, amounting to a total of 25 points.

#### Quantification of the MEE as part of the intake questionnaire

To quantify the MEE we used information gathered from the intake questionnaire showing insight into the participants’ exposure to an enriched environment. The decision to use the findings of the intake questionnaire was based on the absence of an inventory that gathers data focusing specifically on a MEE for young children between the ages of 5–10.

**Fig 1:**
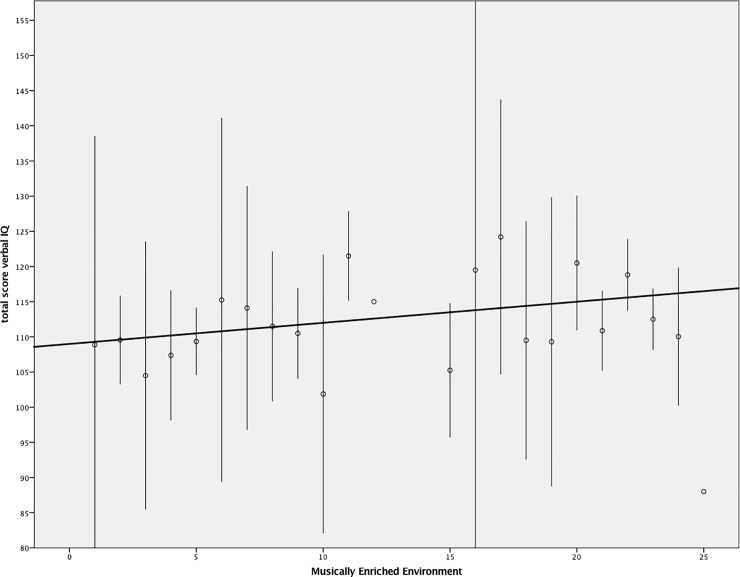
Distribution of individual WISC-III scores in relation to a MEE. Fig 1 shows the distribution of intelligence scores compared to participants across different gradations of experiencing a MEE as computed by the regression model. Vertical lines represent distribution across participants. The diagonal line depicts the differences in predicted values between the MEE intake questionnaire score and verbal intelligence as measured by the WISC III.

The subsection on a MEE has been divided into five main sections, i.e. music lesson, preferences, exposure, activity and parents’ use of music. Each section consists of several questions and is quantified by yes (= 1) and no (= 0) answers and a 5-point Likert scale (see Table 1). Numerical representations of the answers were added together to yield a total score between 0 and 25; i.e. section 1 on music lessons: total score 3; section 2 on music exposure: score 5; section 4 on music activity: score 1; and section 5 on parents’ use of music: score 16. Overall, they yield a maximum score of 25 points. Additionally, descriptive information was used to determine the genre of music and the type of musical instrument, this data was not represented numerically and serves as additional information. To be able to determine whether a child was exposed to an enriched environment a threshold was set at the halfway mark, i.e. 12.5 points, according to standardised statistics literature in clinimetrics [30]. Scores below this threshold describe exposure to a MEE to a lesser extent, and scores above 12.5 describe exposure to a greater extent (Fig 1).

As the items were designed to give insight into the exposure of a MEE and formed part of a larger intake questionnaire (Cronbach alpha 0.79), we investigated the mean inter-item correlation, yielding 0.31, therefore operating in the recommended optimal range of 0.2 to 0.4.

#### Neuropsychological test battery

The following EsF were tested: planning (Tower of London), working memory (Klingberg task), and inhibition (Go/no-Go task). Additionally, verbal IQ was assessed with four sub-tests (I, III, V, VII) of the WISC-III as well as short term memory using the Klingberg matrix. These tests were administered using an iPad 2 running iOS 7 or higher (screen size 9.7 inch, 1024 x 768, 132 ppi, multi-touch). This minimises interaction and influence of the researcher as s/he stands behind the participant during testing instead of in front of the participant actually reading or showing the test trials, as may be the case in traditional ‘pen and paper’ tests. All cognitive tests used were amended from the common ‘pen and paper’ tests and were coded in Apple script and Apple Developer Xcode 6 software by professional programmers together with the authors. As this study describes the baseline measure of a longitudinal study [24], test-retest validity is based on available literature and the handbooks of each individual test.

#### Planning: Tower of London (ToL)

Planning was tested with the Tower of London test (ToL) [31], which was shown to be a valid measure of higher-order problem solving. Participants perform several trials by sorting coloured balls to match the provided final constellation of the balls in as few moves as possible. The balls must be shifted on three rods and can only be moved from rod to rod one at a time. Time to complete the task as well as number of moves to achieve the goal are measured. Test-retest reliability of the ToL is adequate at r = 0.739 and 0.734 [32]. As there is no suitable scoring method for the ToL in the literature, which allowed more subtle differences in scoring rather than simply correct or incorrect, the authors composed a refined scoring methodology [24]. The score is based on the maximum points to be obtained per trial, respectively one point for the three-ball problem, two points for the four-ball problem and three points for the five and six ball problem. Each excess move (moves which are more than the minimum moves necessary to solve the trial), are subtracted from one another and multiplied by ten to yield an excess error percentage. This percentage is subtracted from the maximum amount of points to be obtained per trial, yielding the overall amount of points, between 0.0 and depending on the trial 1.0, 2.0 or 3.0.

The sum of all trials yields the final score, which can reach a maximum of forty points. Points can be achieved on equal distances from 0.0 to 40.0, with stepwise .1 increase or decrease, yielding a much more precise scoring [24].

#### Klingberg short-term and working memory task

Visuo-spatial short-term and working memory is measured using a dot matrix, whereby participants must select dots which appear in a four by four grid in forward (short-term memory) and reverse order (working memory) [33,34]. The task involves remembering the location of the dots and increases consecutively in difficulty (more dots must be remembered). Test-retest validity and reliability correlates with the components span forward and span backward of the test, r = 0.79 [35,36]. Each level represents four trials with an increasing number of dots (Level 1 = 4 x 2, Level 2 = 4 x 3, Level 3 = 4 x 4, Level 5 = 4 x 5 and Level 6 = 4 x 6). Multiplying the correct trial times by the level (1–6), was included into the algorithm, as our version divided the total amount of trials with the overall reaction time. This number, however, did not represent a correct value and had to be multiplied by the number of levels to correct for the total reaction time resulting in a corrected final value for each participant.

#### Inhibition: Go/no-Go task

Inhibition was assessed using a Go/no-Go task [37] which measures the ability to inhibit a motor response to a presented visual stimulus. In one outcome, the participant is required to perform a response (go condition) and withhold a motor response when the object is crossed through (no-go condition). Accuracy and reaction time are measured to indicate the participants’ inhibitive qualities.

The version used here was created specifically for children and depicts an aeroplane which flies either left or right (go condition, pressing either the left or right button respectively). If the plane is crossed through the respondent should not press the left or right button on the screen. A latency time of 10ms is added to the cross (the cross appears later than the plane) when the participant performs better on the no-go stimulus adding difficulty to each trial. Equally, 10ms is subtracted when the participant performs worse on the no-Go-stimulus to decrease difficulty, motivating the participant to continue and finish the task. Test-retest coefficients are satisfactory for the action inhibition condition (no-Go) Mean probability of inhibition r = 0.72, Mean reaction time (MRT) r = 0.66, Total Errors r = 0.49, Slope of inhibition function r = 0.32 and Standardised Reaction Time SSRT r = 0.21. To determine the final stop signal reaction time, the total amount of stop signal delay time (SSDT) (as delay times vary per trial, the amount of delay was different per participant amounting to an average stop signal delay time: added or subtracted 10ms) was subtracted from the mean reaction time (MRT) and divided by the percentage of errors (= noGo condition). SSDT subtracted from MRT resulted in a raw score, which was corrected for errors, resulting in a standardised Stop Signal Reaction Time. The level of inhibition was determined through an error percentage corrected for *standardised* SSRT, whereby a lower SSRT indicated a better ability to inhibit during the stop stimulus. This approach was chosen as a more conservative scoring that may have produced high accuracy scores. However, low processing speed may have resulted in low efficiency scores [38,39].

#### Verbal intelligence

Sub-tests of the Wechsler Intelligence Scale (WISC) for Children 3^rd^ Edition (WISC-III) [40] were used to measure verbal intelligence. Sub-tests I (Information), III (Similarities), V (Verbal Comprehension) and VII (Arithmetic) were administered.

The selection of the short-form of the WISC-III-NL is based on acceptable reliability and validity as divided into the subtest vocabulary (α = 0.96, r = 0.85), similarities (α = 0.93, r = 0.81), mathematical skills (α = 0.93, r = 0.74) and information (α = 0.95, r = 0.82) showing highest correlations with the total verbal intelligence scale compared with other subtests of the WISC-III-NL [40]. The sum of the correlations between the subtests (Σ_rjk_ = 3.41) gives the constants a = 1.5 and b = 40. As described in the WISC-III manual, mathematical skills are presented as verbal problems in this sub-test of the WISC-III and therefore, are part of verbal intelligence measures [40].

Verbal intelligence was chosen over a whole scale or a performative intelligence as education methods in Dutch primary schools are based on verbal problem-solving in both language and mathematical skills. The WISC-III was chosen above the WISC-IV as validity scores are more reliable for the subtests as used in this study.

### Data analysis

We tested whether mean scores on the MEE would show a significant relationship between EsF (planning, working memory, and inhibition), short-term memory and verbal intelligence. Scores on EsFs, short-term memory and sub-tests of the WISC-III were converted into z-scores and, according to factor analysis, summed up to define specific domains. Although the research question concerned a relationship between MEE and overall cognitive functions, which include executive sub-functions (planning, working memory, and inhibition), short-term memory and the WISC-III sub-tests, we investigated whether we could combine variables into domains before entering them into the regression model. In order to do so, we have included executive sub-functions (planning, working memory, inhibition), short-term memory (Klingberg forward condition), and verbal intelligence into one overall domain. The inter-item reliability analysis yielded a Cronbach’s alpha that was, however, too low: 0.58 to use all variables at once for regression. In the second step, we have attempted to construct the domain ‘executive sub-functions’ (planning, working memory, and inhibition) to control for multiple tests. The inter-item reliability Cronbach’s alpha was, however, too low: 0.61. Therefore, we have discarded both domains and entered each test step-by-step into our regression model. We tested the hypothesis whether an exposure to a MEE is a significant predictor to EsFs, short-term memory or verbal intelligence (step 2) after controlling for age and parents’ education (step 1).

To perform the above-mentioned analyses, SPSS Statistics and R statistical software were used (SPSS 24, IBM and R Language 3.3.2.). As we could not construct an ‘executive functions’ domain nor a ‘cognitive function’ domain, we used steps 1 and 2 for each EsF, short-term memory and the short version of the WISC-III. The level of significance was adjusted using a Bonferroni correction. Levels of significance therefore were adjusted from *p* ≤ .05 to *p* ≤ .01, to control for multiple tests used in our statistical model.

## Results

176 children completed the measurements. Preliminary analyses were conducted to ensure no violation of the assumption of normality, linearity, multicollinearity and homoscedasticity.

The ages within the sample represented the usual age distribution in Dutch primary schools for grades 3 and 4 at baseline. A chi-square analysis has shown no statistically significant difference between age at the time of measurement χ^2^ (1) = 1.61, p = 0.784.

Parents’ education and age of the participants were used as additional factors in the regression model, not influencing the predictor as set at experiencing a MEE (Table 2 for details). The MEE score was computed based on the music related intake question (Table 2) within the Regression model analysis.

After the entry of WISC-III (Verbal Intelligence: VI), ToL (planning), Klingberg Forward (short-term memory), Klingberg Visuo-spatial Backward Span (Working Memory), and Go/no-Go (Inhibition) at step 2 respectively, the total variance explained the model on verbal IQ. It did not account significantly on measures of each of the EsFs and short-term memory (Table 3).

**Table 3:** Predictions of executive functions: Results of linear regression analysis.

Dependent variable	Predictor	*β*	*sig*	*R*^*2*^
**Planning (ToL)**				
step 1	age	.142	.10	
	education mother	.045	.64	
	education father	.042	.64	
step 2	MEE	.035	.06	.468*^,^ ^a^
**Inhibition (Go/noGo)**				
step 1	age	.092	.08	
	education mother	.051	.39	
	education father	.052	.32	
step 2	MEE	.132	.06	.366*^,^ ^b^
**Working Memory (KL-Matrix)**				
step 1	age	.184	.03	
	education mother	.150	.12	
	education father	.201	.04	
Step 2	MEE	.131	.08	.312*^,^ ^c^
**Short-term Memory (KL-Matrix)**				
step 1	age	.189	.02	
	education mother	.060	.53	
	education father	.069	.48	
step 2	MEE	.08	.09	.323*^,^ ^d^
**Verbal IQ (WISC-III)**				
step 1	age			
	education mother	.009	.49	
	education father	.009	.48	
step 2	MEE	.285	.02	.326**^,^ ^e^

Edu = educational level, β = standard coefficient Beta value, sig. = statistical significance, *R*^*2*^ = model variance Predictions.

* > .01

** < .01

^a^ *F* (4, 172) = 18.933

^b^ *F* (4, 172) = 19.31

^c^ *F* (4, 172) = 23.21

^d^ *F* (4, 172) = 23.21

^e^ *F* (4, 172) = 36.23

However, when computing the model with experience of a MEE and verbal IQ (variance 32.6%, controlled for age and parents education level), a positive trend between an enriched environment and verbal intelligence scores, β = 0.285 (*p* = 0.02, Table 3; Fig 1), was revealed.

## Discussion

The primary goal of the present study was to examine whether a relationship exists between a MEE and executive sub-functions (planning, working memory, inhibition), short-term memory, and verbal intelligence. The results show only a trend (*p* = 0.02) concerning the relationship between MEE and verbal intelligence. No significant effects were observed between MEE, EsFs, and short-term memory.

We first address the negative findings. The lack of a relationship between MEE and specific EsFs (planning, working memory, inhibition) has been observed before [21]. In that study, there was no relationship between musical sophistication (formal music lessons) and cognitive flexibility, measured by a switching task. Participants’ musical experience consisted of formal musical training (in fact a more intense musical training compared with our more informal MEE). In contrast, Slevc and colleagues found a positive relationship between MEE and working memory [21]. They argue that it is the formal musical training itself that requires working memory, which is why they found a relationship between musical experience and scores on the working memory task. In other words, our MEE, consisting of attending concerts, listening to music or music theory lessons at school, for example, may not have been specific enough, meaning that the aspects of our MEE did not ‘train’ specific EsFs such as planning, working memory, and inhibition. The same line of reasoning may hold for the lack of relationship between MEE and short-term memory. Indeed, in one of our own recent studies, children showed an improvement in planning and inhibition, in addition to verbal intelligence. In that longitudinal study, children participated in a more intense music programme, including playing instruments, listening and music theory, across a period of 2.5 years (for more details see [24]). Furthermore, it has been observed that playing an instrument may have a beneficial effect on both planning and inhibition (impulse regulation) [41].

Interestingly, exposure to our MEE revealed a positive relationship with verbal intelligence. As this relationship showed a trend (*p* = 0.02), one should consider this finding with caution. The relationship between music and language has been investigated for decades as reflected, among others, in the elegant work of Patel [42] or Peretz [43]. Various studies have confirmed a relationship between music listening and language as they share similar capacities in terms of perception and processing as well as neuroanatomical markers in language aptitude and musicality [41–48]. For example, listening to either music or language recruits similar brain structures, e.g. the arcuate fasciculus [45–47] or the Heschel’s Gyrus and Broca’s area [44,48] and therefore supports a possible enhancement of verbal intelligence scores when experiencing music [44–48]. Additionally, multiple studies on the influence of music on sub-components of language e.g. semantics, spelling, syntax and vocabulary have supported the relationship between music listening and language [12–14,44–49]. These studies argue that music and language share capacities such as rhythm and melody and therefore overlap with associated neural networks, supporting a possible transfer [24,44–50]. Further support for this mutual influence emerges from the available literature [51–54]. Yang et al. [54] found a positive effect of music on second language acquisition. In that study, testing second language only appealed to listening comprehension, knowledge of phonology, and word meaning whereas testing first language appealed to a wider range of language functions [54]. They argue that the fewer components of language one assesses, the higher the chance that one will find a beneficial effect of music on those components. Dawson et al. [55] found a positive relationship between pitch discrimination and musical experience. These results suggest that informal music exposure may indeed enhance music-related sound features in first language understanding and modulation. Even in very young children, ‘informal musical experience’, e.g. singing and playing a rhythm to music, made them more sensitive to detecting a deviant tone in a series of standard tones [29]. In other words, informal musical experience improved the quality of auditory processing [29,55,56].

### Limitations

A first limitation is that the items included in our MEE have not been validated. We could have used the scale for musical sophistication, developed by Müllensiefen and colleagues [57]. This scale is a comprehensive questionnaire that focuses on the general adult population through self-report. The questionnaire requires that the participants read and write about music, activities that are usually not performed by children ranging between the ages of 5 and 10 years. The latter group of children participated in the present study, and therefore Müllensiefen’s GOLD-MSI approach was inappropriate [57].

A second limitation might be that the present study is a cross-sectional study, implying that one can only report relations instead of *causal* relationships. Although we found non-significant relations (we adjusted the level of significance to control for multiple tests), we reported that the relationship between MEE and verbal intelligence showed a trend (*p* = 0.02), which can be explained by the strong explorative character of the present study. Nonetheless, as this study has investigated a cohort and neither used a pre- post measure nor a control group, we do not state any effect from MEE to cognitive performance. Yet, being exposed to a musically enriched environment, performs on the edge between active listening, active engagement and passive listening [58]. Visiting a concert, invites the listener to engage with the music, listening with focused attention. Passive listening, such as in background music when studying or playing with peers, spontaneously engages related language networks in the brain, allowing for a possible association between a MEE and verbal intelligence scores [47].

However, to further consolidate such a trend, validation as well as expansion of the questions of the MEE should be taken into consideration. Giving the need to be able to measure the exposure of a MEE in young children, would therefore increase our knowledge of the benefits of either learning to play an instrument [24] or the exposure to an enriched environment significantly [29, 55].

## Conclusion

In conclusion, the present results suggest, that a MEE does not predict increased scores on executive sub-functions, but may have a positive relationship with verbal intelligence. As there are no comparable cross-sectional studies examining children in their first years of development, further investigations have to tackle the question whether a MEE can predict higher cognitive functions besides verbal intelligence.
